# China's space program on the march

**DOI:** 10.1093/nsr/nwaf335

**Published:** 2025-09-05

**Authors:** Mu-ming Poo

**Affiliations:** Scientific Director, Institute of Neuroscience, Center for Excellence in Brain Science and Intelligence Technology, Chinese Academy of Sciences, China Executive Editor-in-Chief of NSR

In May 2025, China successfully launched the Tianwen-2 asteroid and comet probe. It will first land on the near-Earth asteroid (469 219) Kamo'Oalewa—a quasi-satellite of Earth—and then proceed to a main-belt active comet 311P/PANSTARRS. Tienwen-2 will collect ∼1 kg of asteroid samples by July 2026, release the sample capsule to Earth via a flyby in late 2027, and then use Earth's gravity to slingshot toward the comet for a 7-year journey—arrival at 311P around 2030 and make observations until 2033. Complementing previous US comet missions, it breaks new ground in small-body exploration by targeting both an asteroid and a comet. This mission is part of the Tianwen (‘Heavenly Questions’ in Chinese) series, which will continue with Tianwen-3, slated for a 2028 launch to collect and return Mars samples to Earth by 2031, and Tianwen-4, an orbital mission to explore the Jupiter System. These missions underscore China's commitment to pushing the frontiers of astronomy and advancing its multi-faceted space programs.

China's lunar exploration program also reached a milestone late 2024 with the Chang’e-6 probe, which collected nearly 2 kg of samples from the South Pole-Aitken Basin on the Moon's far side. These samples have revealed striking asymmetry between the Moon's near and far sides. Research teams led by Chinese Academy of Sciences (CAS) institutions including the Institute of Geology and Geophysics, the National Astronomical Observatories, and the Guangzhou Institute of Geochemistry, along with Nanjing University and others, have recently published their findings in a series of three articles in *National Science Review* (https://doi.org/10.1093/nsr/nwae328; https://doi.org/10.1093/nsr/nwaf103; https://doi.org/10.1093/nsr/nwaf087) and four articles in *Nature* (https://doi.org/10.1038/s41586-024-07639-w; https://doi.org/10.1038/s41586-024-07640-3; https://doi.org/10.1038/s41586-024-07641-2; https://doi.org/10.1038/s41586-024-07642-1). Major findings include the formation time of the Moon's oldest crater, the evidence of prolonged volcanic activity, the fluctuation of the ancient Moon's magnetic field, the weathering and ‘ultra-depleted’ mantle of the Moon's nearside, and its lower water content of the Moon's far side than the nearside. These discoveries provide new insights into the Moon's geological evolution and pave the way for a deeper understanding of planetary formation.

One of the most ambitious projects in China's space program is the International Lunar Research Station (ILRS), led by the China National Space Administration in collaboration with Russia's Roscosmos and other international partners. The ILRS aims to establish a long-term robotic and eventually crewed lunar base by the mid-2030s. Key steps for 2026–2030 include the Chang’e-7 mission in 2026, which will conduct a comprehensive exploration of the lunar south pole, using an orbiter, lander, rover, and a ‘mini-hopping detector’ to identify potential ILRS sites with sunlight and water resources. This will be followed by Chang’e-8 in 2028, testing technologies for autonomous construction, such as using lunar regolith to manufacture bricks and structures, closed-loop life support systems, and robotic infrastructure. Heavy-lift launchers like the Long March 10 rocket will deliver modules that include energy and communication hubs (solar arrays and relay satellites), resource mining rovers (for extracting water ice and processing regolith), and a remote-controlled science lab for experiments. By 2030, the core ILRS infrastructure is expected to be operational. Crewed missions for ILRS assembly and maintenance are planned for the early 2030s, with expanded infrastructure including greenhouses, radiation shields, and research labs. Advances in artificial intelligence (AI) may also lead to the emergence of highly intelligent ‘astrobots’ capable of most tasks on the ILRS, reducing the need for prolonged human presence.

The rapid advancement of AI technology is reshaping visions of space exploration. Robotic missions are likely to dominate solar system exploration in the future. Humans are not evolved to live in places other than the Earth and how normal human physiology and cognition could be maintained for long residence in space remains unclear. Astrobots with advanced intelligence and behavioral adaptability are likely to be produced within a decade. They could be trained with a world model that adapts effectively to working and learning in designated space environment while operating under strict Earth-based human oversight.

China’s space program emphasizes international collaborations. For instance, Chang’e-5 lunar samples have been shared with six countries, including the US and France. A newly established International Deep Space Exploration Association (IDSEA) aims to involve developing nations in CubeSat projects, and foreign astronauts are currently under training for future space station missions. Such collaborations will demonstrate how nations could work together not only to address global problems on Earth but also to achieve humanity's shared goals in understanding the universe.

